# Analysis of Dynamic Changes in Sedimentation in the Coastal Area of Amir-Abad Port Using High-Resolution Satellite Images

**DOI:** 10.3390/jimaging11030086

**Published:** 2025-03-18

**Authors:** Ali Sam-Khaniani, Giacomo Viccione, Meisam Qorbani Fouladi, Rahman Hesabi-Fard

**Affiliations:** 1 Department of Civil Engineering, Babol Noshirvani University of Technology, Shariati Ave, P.O. Box 48, Babol 47148-71167, Mazandaran, Iran; ali.sam@nit.ac.ir (A.S.-K.); rahmanhesabifard518@gmail.com (R.H.-F.); 2 Department of Civil Engineering, University of Salerno, 84084 Fisciano, Italy; 3 Department of Civil Engineering, University of Science and Technology of Mazandaran, Behshahr 48518-78195, Mazandaran, Iran; m.qorbani@mazust.ac.ir

**Keywords:** longshore sediment transport, high-resolution satellite images, shoreline change prediction, support vector machine, artificial neural network, lifetime, Amir-Abad port, Caspian Sea

## Abstract

Sediment transport and shoreline changes causing shoreline morphodynamic evolution are key indicators of a coastal structure’s operational continuity. To reduce the computational costs associated with sediment transport modelling tools, a novel procedure based on the combination of a support vector machine for image classification and a trained neural network to extrapolate the shore evolution is presented here. The current study focuses on the coastal area over the Amir-Abad port, using high-resolution satellite images. The real conditions of the study domain between 2004 and 2023 are analysed, with the aim of investigating changes in the shore area, shoreline position, and sediment appearance in the harbour basin. The measurements show that sediment accumulation increases by approximately 49,000 m^2^/y. A portion of the longshore sediment load is also trapped and deposited in the harbour basin, disrupting the normal operation of the port. Afterwards, satellite images were used to quantitatively analyse shoreline changes. A neural network is trained to predict the remaining time until the reservoir is filled (less than a decade), which is behind the west arm of the rubble-mound breakwaters. Harbour utility services will no longer be offered if actions are not taken to prevent sediment accumulation.

## 1. Introduction

The northern Iranian coastline extends for approximately 820 km from Turkmenistan’s western border to Azerbaijan’s eastern one. As one of the key ports in the region, Amir-Abad is strategically located on the southeastern coast of the Caspian Sea, hosting international commercial vessels. A noticeable challenge that port managers face is the accumulation of sediment in conjunction with some irregular events, which has altered the region’s hydrodynamic pattern. Hence, supervisors have determined that parts of the port’s basin and surrounding areas should be dredged intermittently to provide the required draft, guaranteeing operational continuity. However, this non-quantified process can only continue for a limited period of time, as it is not feasible to sustain it indefinitely. The motivation is twofold: First, existing structures have a limited penetration depth, and their stability will be questioned when this criterion is reduced. Second, dredging is a costly process, as it involves expensive operations, interfering with normal port activities. Therefore, it is essential to accurately quantify changes in the shoreline position and the shore area surrounding the port.

Various methods exist for quantifying sediment transport rates and predicting coast configurations over time [1,2,3]. In general, computational methods can be categorised into the following two classifications: empirical methods and math-based methods. The CERC formula [4] is an appropriate and well-known empirical method. The empirical coefficient plays a significant role in sediment transport rate estimations when using this relation. The SPM code recommends a value of 0.39, which does not take into account the specific conditions of each area and is based on a study by Komar and Inman [5]. According to Fowler et al. [6] and Wang et al. [7], the CERC relation can only be used to calculate sediment load orders when the empirical coefficient has not been calibrated with precise data. Thus, they argued that the CERC formula can be used to predict potential sediment transport rates that are two to five times greater than dredging records, bypassing rates, or volumetric variations. In contrast, Miller [8] asserted that if the empirical coefficient is not calibrated, it may underestimate sediment transport rates. Using the CERC formula, Samaras and Koutitas [9] found that the evaluated longshore sediment transport rates were higher than those obtained by Kamphuis’s [10] and Bayram et al.’s [11] formulas. Hence, it can be concluded that, in each region, there is a direct correlation between the accuracy of the evaluations and the experiences of users of empirical formulas, which can lead to unrealistic assumptions and higher project costs.

Mathematical methods may be a better, more viable alternative. In academia, several researchers have either applied or developed numerical methods as a means of overcoming empirical restrictions or analytical simplifications, and a variety of methods have been formulated for this purpose. Three types of models can be categorized based on the following spatial dimensions: one-dimensional, two-dimensional (horizontal (H) or vertical (V)), and three-dimensional (full) models. The simplest simulation involves a one-dimensional model of sediment transport along shorelines. Afterwards, two-dimensional models can be divided into horizontal or vertical solution domains. In 2DH models, the flow values are integrated vertically, and gridding is conducted horizontally. The 2DV models, on the other hand, do not consider variables along the shoreline and perform gridding on a vertical plane. Accordingly, Wang [12] used a depth-averaged model to simulate sediment transport in the curve of a channel with fixed walls. To describe the cross-stream distribution of the flow intensity located at the channel curves, a semi-empirical relationship is proposed. Effects such as the bed load transport angle were practically neglected. It is generally accepted that two-dimensional models constrain simulation processes compared to real-world conditions due to the assumption of a constant distribution pattern for governing variables in one particular direction. In an attempt to overcome the limitations of two-dimensional models and to obtain accurate modelling results, scientists have developed three-dimensional models where sediment transport is coupled with CFD solvers. Sakhaei and Niksokhan (2021) [13] examined the pattern and rate of sediment transport around Amir-Abad port using the MIKE model. The solution domain of their study was considered such that the effects of structures around ports on sediment transport during port functions were not considered. The Delft3D model was used by Mayerle and co-workers [14] to study sediment transport along three dimensions. Their study aimed to improve the Delft3D’s accuracy by calibrating the hydrodynamic information using measured data. It should be mentioned that the use of three-dimensional models is not without complications. Firstly, the lack of precise predictions of wave data or sediment properties (during the design process, as well as during operation) in a particular region has a significant impact on the accuracy of the results. Also, the relationships and computations are based on the assumption of an infinite sediment source. However, local and timely variations due to unpredicted occurrences in these models are not taken into account. Moreover, the non-uniformity of portions of a shoreline affects the potential transport rate. The breaking wave height, angle, and shoreline orientation of these shore types need to be calculated accurately. Hence, tools for ray-tracing, as well as performing grid-based wave refraction analysis are required [15,16]. In the 3D-modelling process, the consideration of all these factors leads to a significant intensification in computational costs.

Mathematical simulations associated with Amir-Abad Port present a number of issues that are not exclusively related to the use of conventional numerical methods. First, the dredging process has been conducted irregularly, as well as without consideration of quantitative computations, leading to the emergence of an unexpected geometric and material pattern that differs from the properties of the natural shoreline. This results in a difference between sediment transport rates over short- and long-terms periods. Additionally, by constructing an industrial yard with 2775 m of breakwaters, which exceed the surf zone in the port upstream, sediment transport mechanisms are affected by interactions between two coastal structures. In the end, the decreasing water level in the Caspian Sea in recent years has changed the shoreline position, in addition to sediment movement and accumulation.

To overcome the computational limitations and quantify changes in coastlines over time, reliable codes suggest the use of remote sensing data. Accordingly, satellite images with high spatial resolution have been used in numerous studies to monitor short- and long-term changes in coastlines [17,18,19,20,21,22]. Monitoring such situations over time has additional benefits beyond the data that may be obtained from satellite images. Extracted remote sensing data can be utilised in conjunction with prediction tools to forecast future events [23,24].

In spite of the existence of many studies with a wide spectrum of methods and algorithms for the prediction of changes in shorelines [17,18,25,26,27,28,29,30,31,32], one of the most promising methods in this field is the artificial neural network [33], which has been used in studies in this field to a limited extent [34,35,36]. In addition to managing and processing a large volume of data with a high degree of nonlinearity, this method provides a high degree of certainty, making it widely used in studies [37,38,39].

Shoreline change in this area is influenced by three simultaneous phenomena. Two of these phenomena are natural, namely, sediment accumulation due to longshore sediment transport and a decrease in the level of the Caspian Sea. Thirdly, there is a phenomenon that is the result of human activity, which involves the construction of a protective structure upstream of the harbour. The simultaneous occurrence of these three phenomena has led to inaccurate, simplified modelling results. Hence, in light of the abovementioned issues, extracted satellite image data can be combined with artificial neural network algorithms to create a reliable framework for longshore dynamic changes, which includes the effects of both natural factors and human decisions. In other words, the coupled model relies on data that allocate these irregular effects. Consequently, first, the current study quantifies and reports changes in the shore area surrounding the Amir-Abad port during its operation through the interpretation of high-resolution satellite images. Second, the shoreline position is digitised and presented at various times from the port’s construction to the present. Eventually, it is attempted to identify patterns in the changes in shoreline position using a trained artificial neural network, for which assuming possible shoreline patterns when the sediment accumulation reservoir is filled, the remaining time until complete port inoperability is estimated.

## 2. Data and Study Area

### 2.1. Study Area

**Figure 1:**
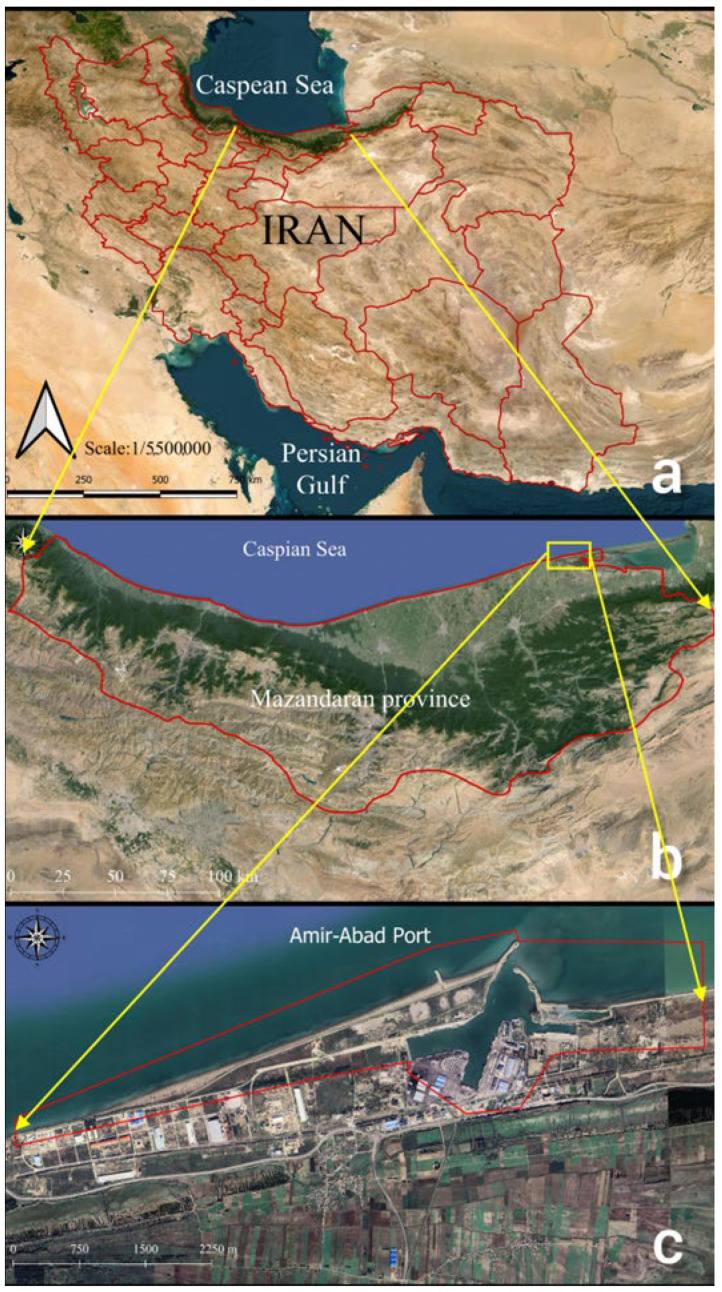
Top-down views of the study areas: (**a**) general overview; (**b**) Mazandaran province, Iran; (**c**) Amir-Abad port in Mazandaran province.

Amir-Abad port is located near the city of Behshahr, Mazandaran province, Northern Iran, south of the Caspian Sea, at a geographical latitude of 36° North and a geographical longitude of 53° East (Figure 1). Its construction started in 1991, and it was established as a free commercial zone. The distance from its western breakwaters to the nearest port, Sadra, a shipbuilding port, situated to the west, is approximately 7.5 km. On the other hand, the nearest eastern port, Turkmen, is located about 60 km from the eastern breakwaters of Amir-Abad port.

### 2.2. Data

The present study utilises high-resolution satellite imagery from the designated region, comprising ten images sourced from the Google Earth Pro (GE Pro) platform [22] at a consistent eye altitude of 3.5 km to analyse the coastal changes that occurred between 2004 and 2023. Additionally, an aerial image with a spatial resolution of approximately 2 m, obtained from the United States Geological Survey (USGS), is included, representing the pre-construction conditions of the port community in 1956. Table 1 provides detailed descriptions of all historical images used in the study, including the source and spatial resolution. The utilisation of these images adheres to the copyright regulations of GE Pro and the EarthExplorer.usgs.gov [40] website. Furthermore, various processes applied to the images were conducted using the free and open-source software QGIS 3.10.14.

## 3. Method of Data Analysis

The high-resolution images need to correspond to the same area and viewpoint. It should be noted that the images available from GE are not provided with ground coordinates. Consequently, the georeferencing of all adopted images prior to their use is required to overlay them sequentially over time, with the aim of detecting changes in the coast of Amir-Abad port.

**Figure 2:**
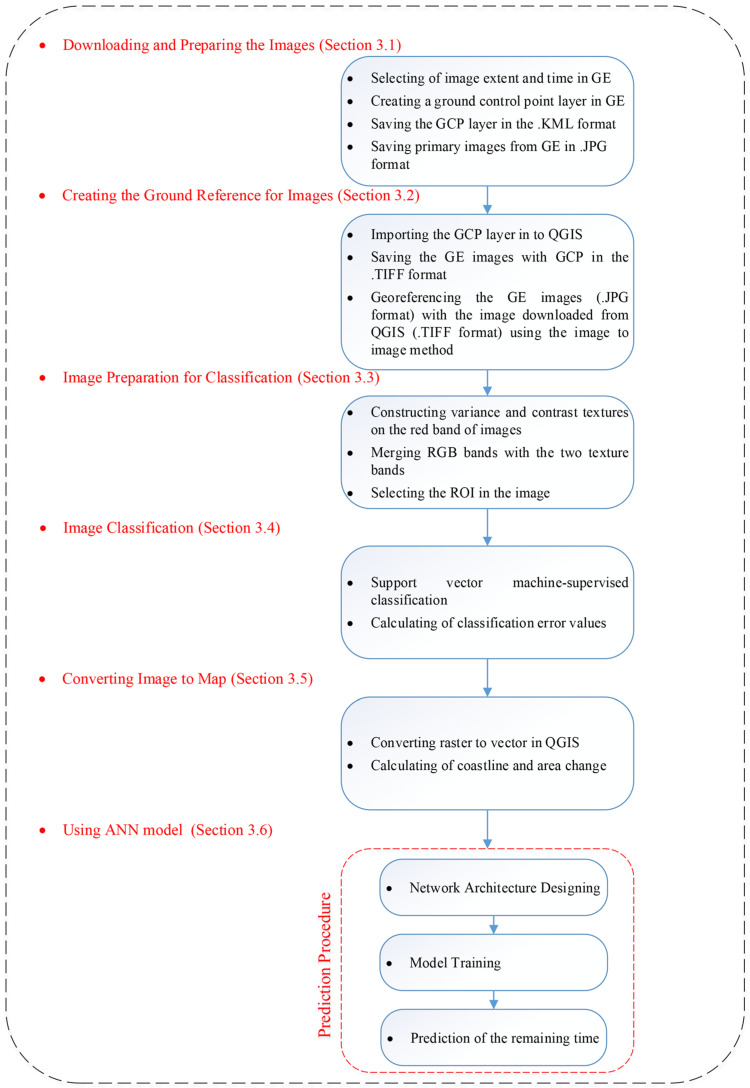
Flow chart illustrating the adopted methodology.

In the next step, the images are prepared for classification by combining the RGB bands with the two texture bands. Then, the satellite images were classified using the support vector machine (SVM) technique to identify the border between land and sea. The next stage involved converting the classified raster images into a vector format so that changes in the shoreline and its advance or retreat can be tracked in QGIS. Ultimately, a neural network model was created using the dates of the images and the numerical values of the shoreline changes to forecast how long it will take for sediment to accumulate and completely fill the area behind the breakwaters main arm. The overall procedure described above is depicted in Figure 2. Specific information on the steps to be undertaken will be given in the next sections.

### 3.1. Preparation of Images

**Figure 3:**
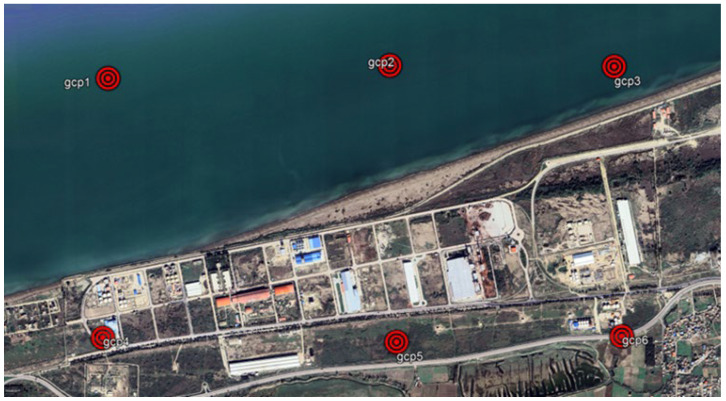
Distribution of Ground Control Points (GCPs) over the study area.

The study area from which the images were progressively captured over time is shown in Figure 3. Six ground control points (GCPs) are identified.

The images (as listed in Table 1) were downloaded using GE Pro 7.3.4.8248 software. The same eye altitude was kept fixed so that the scale was the same for all of images. The coordinates of the ground control points (GCPs) were obtained as a KML (Keyhole Markup Language) file, for use in the QGIS 3.10.14 software from GE Pro 7.3.4.8248.

### 3.2. Image Georeferencing

Before downloading the images, the study area and the time intervals between images captures were selected first and, as mentioned in Section 2, the images were downloaded via GE Pro 7.3.4.8248 software and from the USGS website, as shown in Table 1. In order to prepare the georeferencing facilities for the images used in GE Pro, before downloading the images, six GCPs with regular distributions, as shown in Figure 3, were selected from the latest available images dated for 2023.11.03 in the study area. When extracting data using the software (GE Pro), the same eye altitude and fixed image frame were used for all images, as shown in Table 1, to ensure that the scale for all images remained constant. Also, the coordinates of the GCPs that were selected from the images dated 2023.11.03 were extracted via GE software as a KML (Keyhole Markup Language) file for loading into the QGIS software.

Images downloaded via GE Pro and from USGS do not come with ground coordinates. To spatially reference these images using the image-to-image georeferencing method, a previously georeferenced image that is similar to the unreferenced image is necessary. The open-source software QGIS 3.10.14, along with the GE Pro plugin, allows users to download georeferenced images in the GeoTiff format. However, this plugin can only access the most recently uploaded image and does not provide access to the history of images available in GE.

Here, an image on 3 November 2023 was acquired using QGIS in the GeoTiff format, which included the necessary control points obtained from GE in the KML format. This image was then used in the image-to-image georeferencing process alongside a similar but unreferenced image that was also prepared from GE Pro on the same date.

Now, we have two very similar images: one without ground coordinates downloaded from GE Pro and the other with ground coordinates obtained from QGIS. The locations of the ground control points (GCPs) are known for both images. Using these shared points, the georeferencing process for each image was conducted with reference to the ground image from 3 November 2023. The image-to-image method has the advantage that the georeferencing accuracy is the same for all of the images, with a root mean square error (RMSE) of 0.1492 units of the image pixels for all images.

### 3.3. Image Preparation for Classification

**Figure 4:**
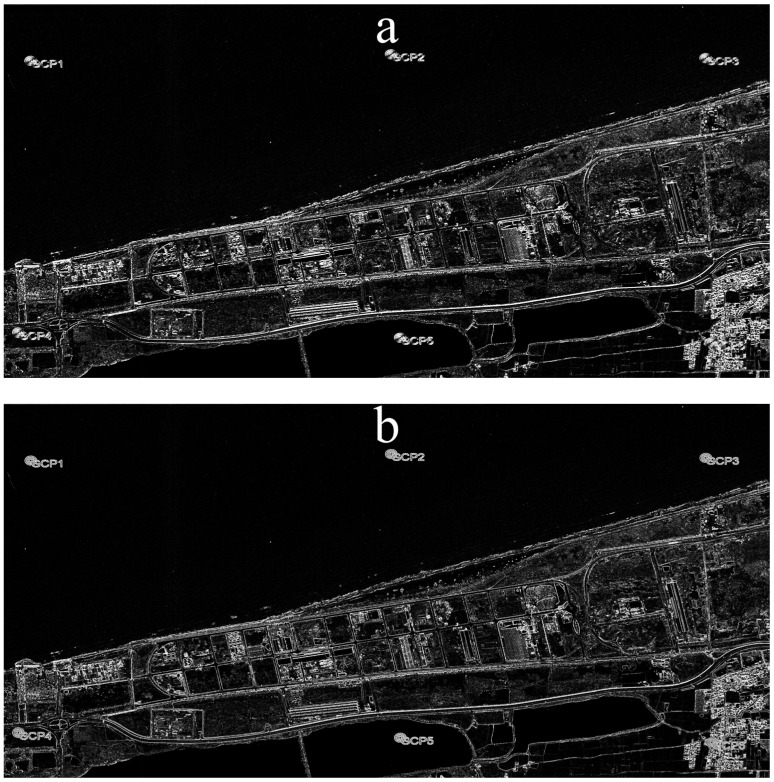
(**a**) Variance texture in the red band of the satellite image; (**b**) contrast texture in the red band of the satellite image. The date of the selected image is 2004.28.05.

After georeferencing, the images must be separated into dry and wet classes. To increase the classification accuracy and highlight the edges of the coastline in the images, first, a variance and a contrast texture are created separately from the red band of each image, which are shown in Figure 4, panels a and b, respectively. The date of 28 May 2004 is taken as an example. 

**Figure 5:**
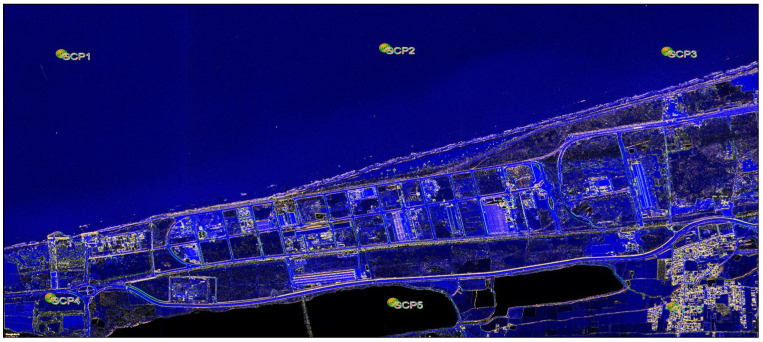
The resulting image ready for classification with the virtual combination of bands 3, 4, and 5.

The textures produced from the original image were ultimately combined with the original image, resulting in a total of five bands in each image. In other words, the first to the fifth bands are the red, green, blue (three principal bands of images obtained from GE Pro), contrast, and variance bands, respectively. As shown in Figure 5, the virtual combination of bands 4, 5, and 3, which, respectively, represent the red, green, and blue colours in the image, achieves a more prominent coastline edge compared to the images given in Figure 4.

### 3.4. Support Vector Machine Classification

**Figure 6:**
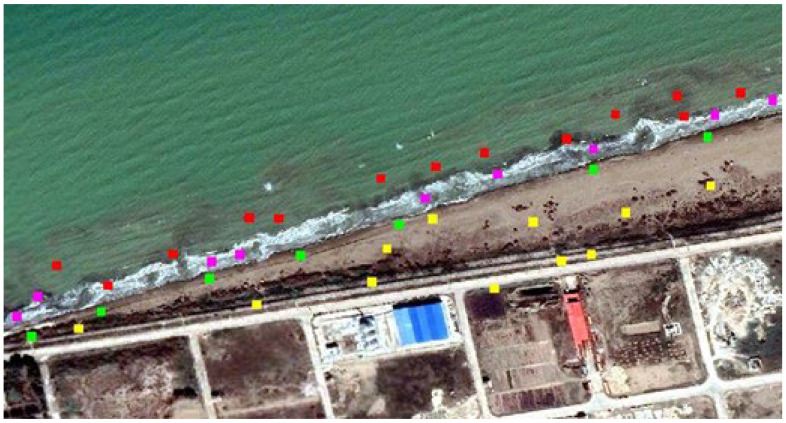
The distribution of the training data in the study area is characterised by red pixels for the water class and yellow pixels for the dry class. In addition, the test data are represented by purple pixels for the water class and green pixels for the dry class.

In supervised classification, the collected training data are of great importance. For each of the water and land classes, 9000 pixels scattered across each image were selected as the training data by the operator (Figure 6). Based on the high-quality GE imagery and field knowledge of the area, pixels that were clearly in the land or water classes were selected as training data. To ensure effective classification control, alongside the training data, represented by the yellow and red points in Figure 6, a total of 4500 land pixels (indicated by green points) and 4500 seawater pixels (depicted as purple points) were utilised as verification points for the land and water classes, respectively. These test points were carefully chosen by the operator from the GEpro images, ensuring they were situated closer to the coastline than the training points to improve the evaluation of the classification accuracy. In total, each image incorporated 27,000 pixels for both training and testing purposes.

Satellite image classification plays a vital role in remote sensing and geographic information systems (GIS). To attain precise classifications, a range of machine learning techniques, including k-nearest neighbours (k-NN), logistic regression, support vector machines (SVMs), and Random Forest (RF), are utilised. k-NN is straightforward to implement and comprehend, establishing it as a reliable baseline technique [41]. It does not rely on a specific data distribution, which can be advantageous. Nonetheless, it may require significant computational resources during the classification process, especially when dealing with large datasets, as it necessitates distance calculations for every training sample [42]. Furthermore, the presence of irrelevant features can adversely affect its performance [43]. Logistic regression offers coefficients that are easy to interpret, facilitating an understanding of the impact of each feature [44]. This method presumes a linear association between the features and the log-odds of the outcome, an assumption that may not be valid in the context of complex satellite imagery data [45]. An SVM is effective in high-dimensional spaces, such as satellite imagery with many spectral bands, and is less prone to overfitting when dimensions exceed samples [46]. Its use of kernel functions enables modelling of the complex data relationships [47]. However, SVMs can be computationally expensive for large datasets, and their performance depends significantly on the choice of kernel and hyperparameter tuning, which can be challenging [42]. Random Forest (RF) is effective in managing noise and large datasets without overfitting, while also providing insights into feature importance [48]. It can be easily parallelised for faster processing. However, RF may underperform compared to support vector machines (SVMs) when the number of features significantly exceeds the number of samples [49]. Additionally, RF models are often seen as black boxes, complicating the interpretation of results compared to linear models.

Satellite imagery is commonly subject to noise caused by atmospheric factors or sensor errors; however, the robustness of support vector machines (SVM) ensures reliable classification even amid such noise. The nature of coastline changes is both complex and non-linear, and SVM’s kernel functions are superior in addressing these complexities when compared to linear models like logistic regression. Considering the limited number of images available for this study and the effectiveness of the SVM technique [50,51,52,53], it was selected for the classification of images aimed at coastline detection. Before classification, principal component analysis (PCA) is used to project the original images onto a new orthogonal coordinate system with lower dimensions. This method separates principal features from dependent features and increases the processing speed.

The radial base function (RBF) kernel demonstrates a significant capacity to distinguish between classes within a multidimensional (e.g., five bands in this study) and intricate nonlinear space. It offers greater flexibility and accuracy in image classification compared to other kernels, such as linear and polynomial kernels [54,55]. Then RBF SVM [53] is performed on individual images. For classification in this way, gamma and kernel function coefficient values between 1 and 0.001 were chosen, as well as a penalty parameter (PP) value between 1 and 1000, to test and determine the error resulting from the classification. In the present study, the most appropriate gamma and kernel function coefficient values were adjusted to 0.200, and the PP value was adjusted to 100. A total of 9000 selected checkpoints from the images of each year, represented by the purple and green pixels in Figure 6, were utilised to create a confusion matrix. This matrix facilitated the calculation of the classification error values employing the SVM method, which includes the overall accuracy and the kappa coefficient for each image. The results are presented in Table 2.

**Figure 7:**
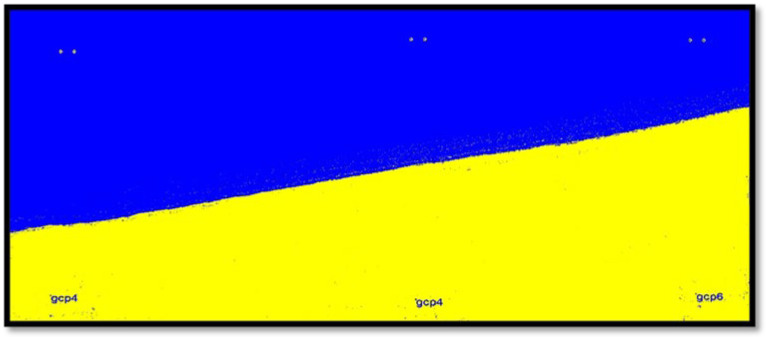
Image related to the year 2004 after the classification and separated into two wet classes in blue colour and dry in yellow colour.

Based on Table 2, the kappa coefficient and the overall accuracy values in the image classification varied in the range of 0.916–0.959 and 92.18–96.90 per cent, respectively. For example, after performing the stated steps, the results of the classification using the SVM method on the part of the image from 2004 is provided in Figure 7.

### 3.5. Convert Image to Map

**Figure 8:**
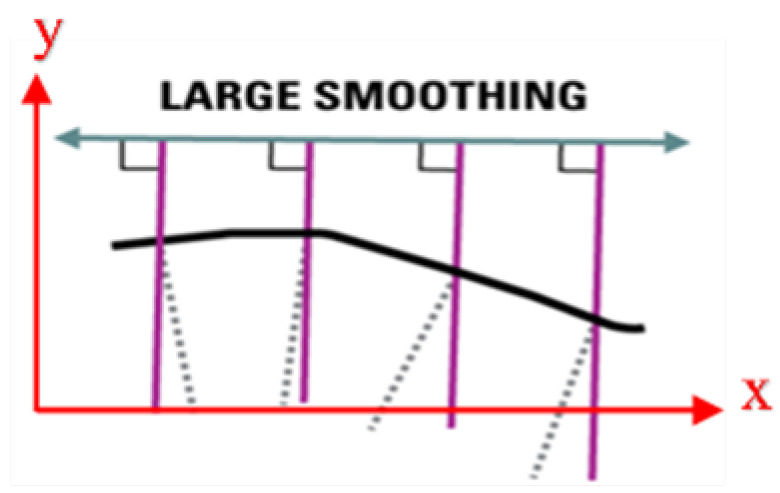
Representation of the large smoothing method for calculating the amount of shoreline change in the local coordinate system.

QGIS 3.10.14 open-source software was used to digitise the images resulting from the classification and evaluate the outcomes of converting the images into polygons and preparing GeoPackage layers. After preparing the polygon of the study’s region, the area and changes in each year were calculated. Also, after defining the coastline in each image, the coastline transgression in a local coordinate system was determined. According to Figure 8, the x-axis in the local system indicates the distance of transgression lines from each other, and the y-axis indicates transgression values calculated using a large smoothing method. In this method, a main line parallel to the horizontal line (x-axis) was chosen as a reference for drawing lines perpendicular to the coastline.

**Figure 9:**
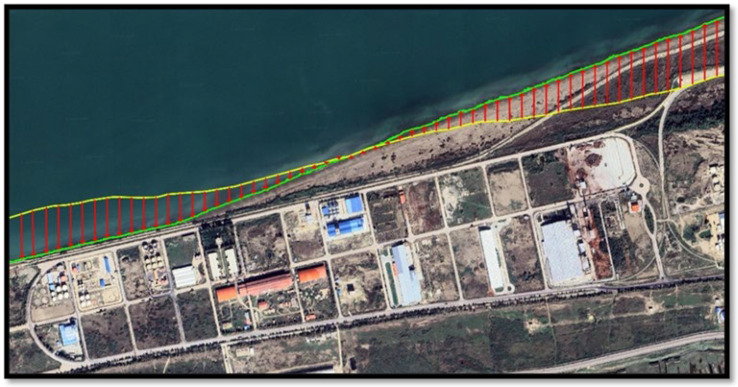
Regression (i.e., seaward shoreline shift) or transgression (i.e., landward shoreline shift) of part of the coastline in the study area between 1956 and 2023.

In Figure 9, lines with red arrows pointing towards north indicate regression, whereas red arrows pointing towards south indicate transgression of the coast between 1956 and 2023. Starting from the left side, the first 100 transgressions are 50 m in distance. Because of the importance of examining the coastline between the main breakwater and the constructed perpendicular arm in 2014, the next 33 transgression lines on the right side were selected at 20 m distances from each other. Perpendicular extension lines were drawn at each point of the coastline so that their Euclidean distances from each other were actually equal. The shoreline in 1956 is shown by the green line, while in 2023, it is indicated by the yellow line.

### 3.6. Projections of Coastline Evolution

This study focuses primarily on the evolution of the shoreline position over time. Several years’ records of shoreline positions are digitised based on a local Cartesian coordinate system. Considering the additional factors that influence the phenomenon of changing shorelines over time (such as changes in water levels, construction of sea structures surrounding the target structure during operation, etc.), the method employed in the present study is significantly enhanced in comparison to other numerical and analytical methods, since it is based on the reality of the governing conditions. The most similar form of shoreline geometry near the main arm of the port is estimated by extracting multiple shoreline versions. Accordingly, each version of shoreline in the assessed zone can be accurately and properly quantified with a regression line. This study defines a catastrophe of normal port operation as the situation where sediment accumulates on the shoreline of the port until it reaches the breakwater tip and enters the port in significant quantities and causes the port to be rendered inoperable. Despite the roundhead being one of the endpoints of this shoreline version, the other end lacks accurate coordinates. Consequently, a number of potential shoreline endpoints are considered. The trained artificial neural network predicts the occurrence time of each of these versions when compared to 1996 (when the mentioned port was constructed).

### 3.7. Artificial Neural Network

Artificial neural networks (ANNs) are paradigms for processing information inspired by the brain. By modifying the nonlinear parameterised mapping between the input and output sets through their neurons, ANNs can be configured and trained, just as the brain employs multiple neurons to process information. A neural network can be applied to problems for which there is no algorithmic solution or where an algorithmic solution would be too complex. An ANN method based on a back propagation learning algorithm was used in this study. In order for a network to meet its target by minimising the mean square error between the actual and predicted vectors, various internal weights and biases are adjusted during the training process. A gradient search technique was used to achieve this minimisation. An appropriately trained neural network can accurately predict outputs for inputs that are not included in its training. Levenberg–Marquardt (LM) is among the fastest and most efficient training algorithms available and, therefore, applied in the current study. In order to provide robustness and stability, it can switch dynamically between Gauss–Newton methods when the problem is well conditioned and gradient descent methods when the problem is far from the solution. As opposed to gradient descent, which is often slow and sensitive to learning rate, and Gauss–Newton, which can be computationally expensive and impractical for large datasets, Levenberg–Marquardt strikes an optimal balance by taking advantage of both second-order derivatives and first-order derivatives. For small- to medium-sized problems, for which a high degree of accuracy is required, Levenberg–Marquardt method is superior to the others because of its flexibility and speed in finding solutions.

**Figure 10:**
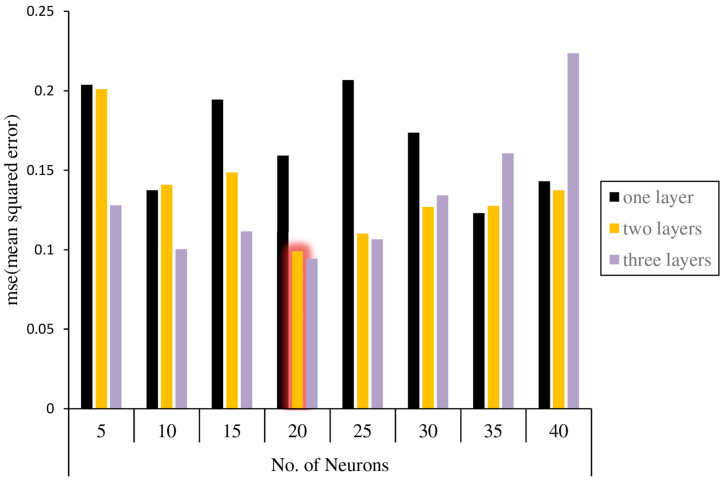
Performance evaluation of the various network architectures.

The number of hidden layers and neurons within each hidden layer are important considerations when designing a neural network. Various types of networks with different architectures are taken into account. Based on the mean square error, the network performance is calculated for each architecture. An analysis of three types of architecture with one, two, and three hidden layers is conducted. Steps for increasing neurons are applied to each of these scenarios. Figure 10 illustrates the results of the network’s performance in each of these network architecture cases. Hence, using a trial-and-error process and checking the accuracy obtained, a network with two layers of 20 neurons is developed.

## 4. Results and Discussion

A number of records extracted from satellite images were analysed in this section with the following three objectives: a—evaluation of changes in shore areas surrounding the port, b—study of changes in shoreline position, and c—prediction of shoreline developments. Therefore, Section 4.1 discusses trends in area changes within different subdomains of the shore zone adjacent to the port. To determine the actual long-shore sediment transport rate, these areas need to be extracted, especially for the early years. According to the CEM code, 40% of the accumulated sediment volume consists of pores between particles and 60% is solid particles. Section 4.2 examines the alterations in shoreline position during the study period. Eventually, in Section 4.2, an artificial neural network is trained to predict how the shoreline would develop based on the extracted data in Section 4.2. In order to determine the expected period of time for the port to be used, this prediction is made.

**Figure 11:**
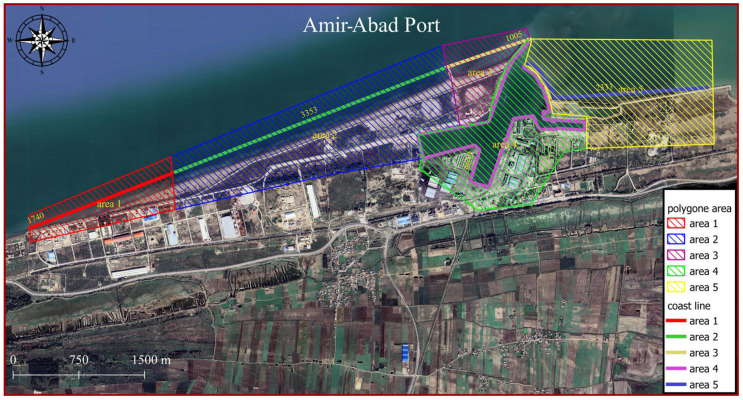
Identification of regions throughout the study area to expedite interpretation and address.

To better deal with the results, the domain surrounding the port is divided into five subdomains (shaded areas in Figure 11). The longshore upstream of the port exhibits two distinct sediment load patterns because of the development of an industrial zone neighbouring the target structure. Region 1 is located downstream of the adjacent industrial area. Because of the lack of sediment in longshore currents, they tend to pick up sediment in this zone. Considering the motion of this flow along the shoreline and the moving of sediment from the seabed, it is expected that sedimentation occurs close to the port (in regions 2 and 3). Due to port managers’ growing concern regarding the formation of shorelines caused by sedimentation phenomenon, during port functions, an embankment structure was realised in the region of sediment accumulation in the reservoir (the following sections explore the efficiency of such choice, on the dynamics of the consequent sediment transport). In the area of sediment accumulation behind the main breakwaters arm, an additional arm has been constructed. Area 2 is located in the sediment accumulation area, which is on the western side of this additional arm. The location between this newly developed segment and the principal arm of the port breakwater is identified as region 3. The term “subdomain 4” refers to the area surrounding the breakwater structure, located primarily on its western and southern sides. Since this subdomain is located in a dry area, no expectations can be made regarding its area change. Region 5 represents the downstream part of the port structure, which is vulnerable to sediment erosion during longshore sediment transport. Port managers constructed concrete walls to prevent sediment depletion in subdomain 5. A storage site was constructed by dredging sand materials from the harbour basin and filling behind this wall. It was not necessary to construct a corrosion prevention structure in this area because the erosion is negligible. 

### 4.1. Examining the Changes in the Shore Area in the Sediment Deposit Region

The coastal conditions in 1956, as defined by the first available aerial image, can be taken as the reference for comparing changes in the shore area surrounding the port. However, this image is not an appropriate option for digitizing because of its lower resolution compared to other images. As a thoughtful solution, since the shore experienced marginal regression or transgression prior to the start of port activities (compared to the 1956 situation) due to human-made actions, the image related to 1996 is therefore taken as a reference. Images from 1956, 2004, 2006, 2014, 2015, 2016, 2017, 2018, 2020, 2021, and 2023 were used to examine the changes in the shore surrounding the port.

Table 3 shows the added/reduced shore area calculated in subdomains 2 and 3. These values are extracted based on the shoreline position in each satellite image.

**Figure 12:**
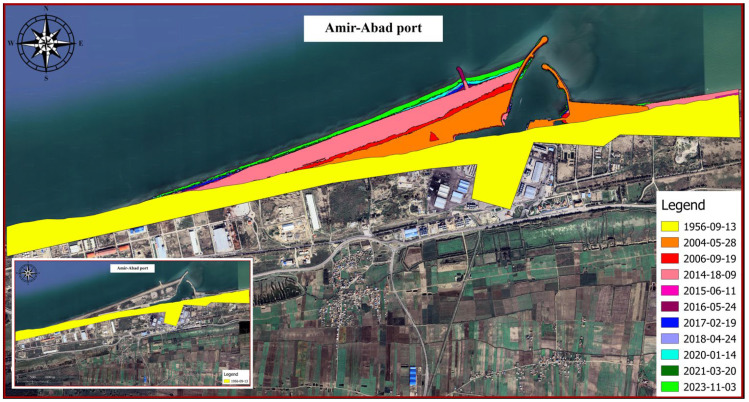
Superposition of changes in the area surrounding the Amir-Abad Port.

**Figure 13:**
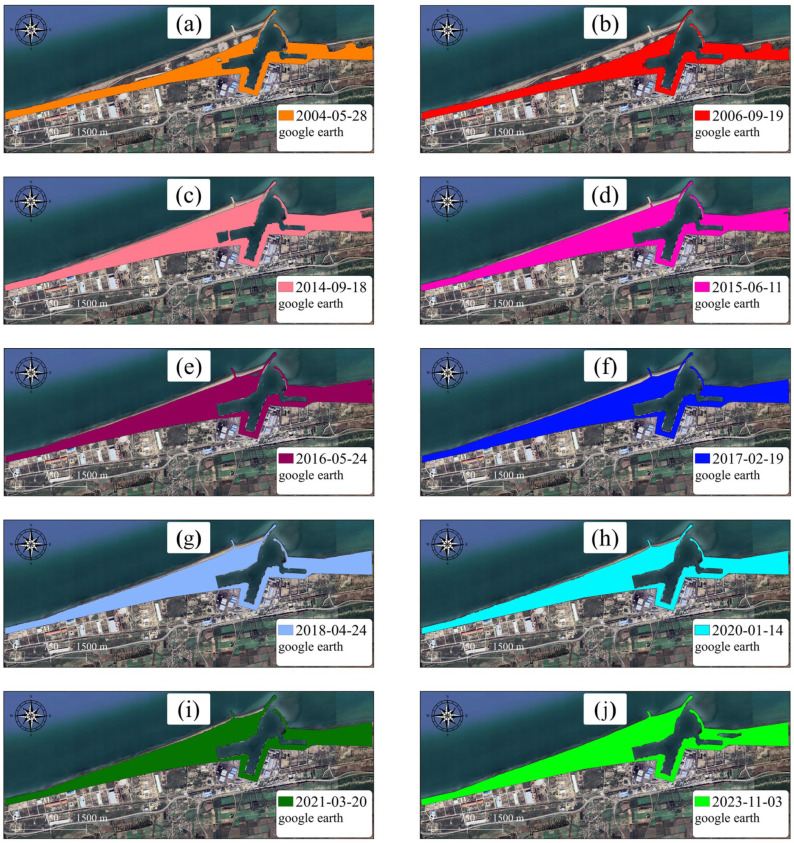
Single snapshots showing coastline evolution, (**a**) the shoreline position in 2004, (**b**) the shoreline position in 2006, (**c**) the shoreline position in 2014, (**d**) the shoreline position in 2015, (**e**) the shoreline position in 2016, (**f**) the shoreline position in 2017, (**g**) the shoreline position in 2018, (**h**) the shoreline position in 2020, (**i**) the shoreline position in 2021, (**j**) the shoreline position in 2023.

According to the extracted data from 1956 to 2014, the shore area has increased overall by approximately 1,099,430 m^2^, reflecting the duration between port operations and upstream industries development. A substantial reduction in the volume of sediment load on the longshore flow has been achieved due to the construction of long breakwaters in the industry region. From 2014 to 2015, the shore at the interface between subdomains 1 and 2 experienced temporary erosion. Therefore, the sum of the sedimentation and sediment removal during this period is overall negative. This negative value indicates that erosion overcomes sedimentation. After leveraging towards new conditions, the longshore sediment load increased again in 2015, leading to shore development. Figure 12 and Figure 13 visualize the changes in the shore zone after the port’s construction. It should be noted that the construction of the upstream industry structure has significantly extended the port’s life. The shore area expansion rate between 1956 and 2014 reached 61,079 m^2^. However, after the implementation of the upstream industrial structure, the shore deposition rate decreased to 49,609 m^2^/y, indicating a 20% drop. It is also worthwhile to note that subdomains 2 and 3 have been affected along a length of about 3.5 km, which corresponds to the length of the sediment accumulation reservoir.

**Figure 14:**
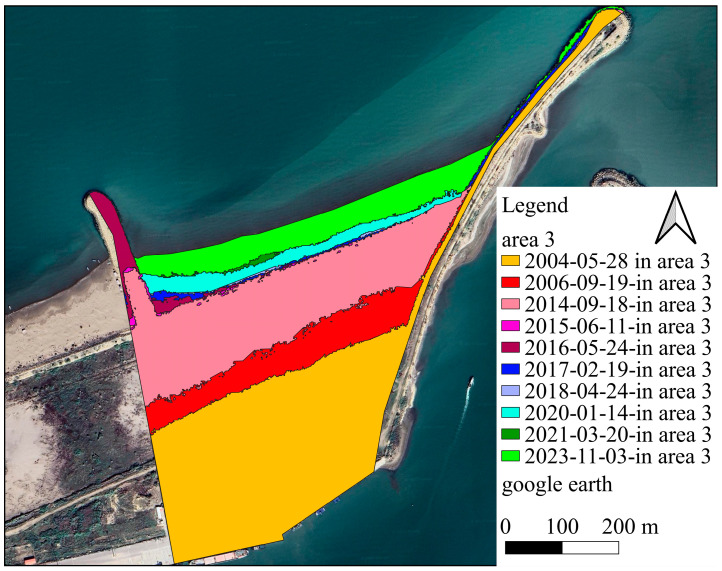
Changes in the coastline in subdomain 3, showing the tendency of the sediment to accumulate along the breakwaters arm.

A further consideration is that in the first years after the port’s construction, the rate of change in the subdomains 2 and 3 areas differs from that in the subsequent years, for various reasons. Such a circumstance is not merely related to the development of the upstream industry. Another reason for this difference is that in the early years of operation, sediment accumulated along the lower length of the western arm. The sediment load had to be directed along the breakwater for a longer distance to escape this region. In the following years, accumulated sediment behind the principal arm of the breakwater left the lower length of the primary arm sediment-free. As a result, sediment has been transported through the port’s entrance and sedimentation in the harbour basin has accelerated. Additionally, sediment flow in the early years of port operation interacted with breakers’ sharp-edged stones. Particles deposited between the structure’s stones and the velocity of sediment-transport flow decreased. Over time, sediment accumulated between breakwater stones, covered sharp edges on stones, and caused the shoreline to direct towards the breakwaters roundhead (Figure 14). By facilitating sediment loading movement along the principal arm, sedimentation in subdomains 2 and 3 was reduced.

Therefore, in subsequent years, the rate of growth in the width of the principal arm has slowed to almost a standstill. It is estimated that most of the sediment that moves along the major arm length enters the port, while the remainder passes through the port entrance and escapes the port downstream.

**Figure 15:**
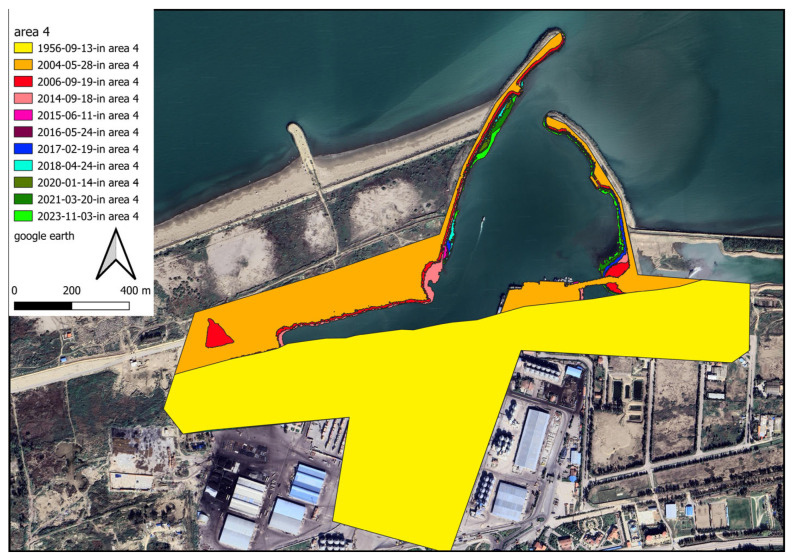
Sediment accumulation inside the stilling basin due to the entrance of the sediment flow.

**Figure 16:**
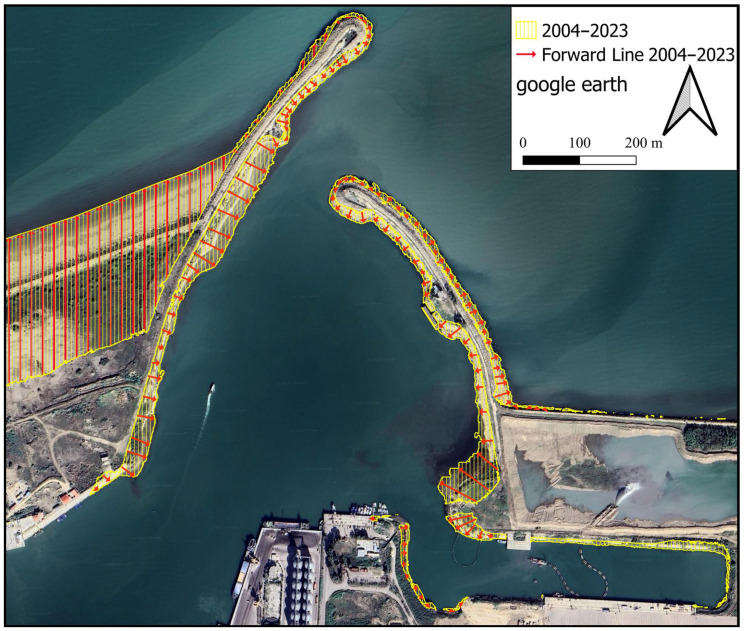
Details of the temporal changes along the edges of the port’s stilling basin for the period 2004–2023.

The sediment load enters the harbour basin following the path outlined in Figure 15 and Figure 16. The sediment loads that enter the basin move in a counterclockwise direction due to the relative reduction in velocity (Figure 16). As an important point to note, sedimentation in the harbour basin cannot be regarded as the result of a decrease in the water level since the required draft of water should be provided for commercial vessels. There is no significant sedimentation on the southern side of the harbour basin, where the concrete wall has minimal friction with the longshore sediment load. In this zone, there is almost no significant sedimentation.

**Figure 17:**
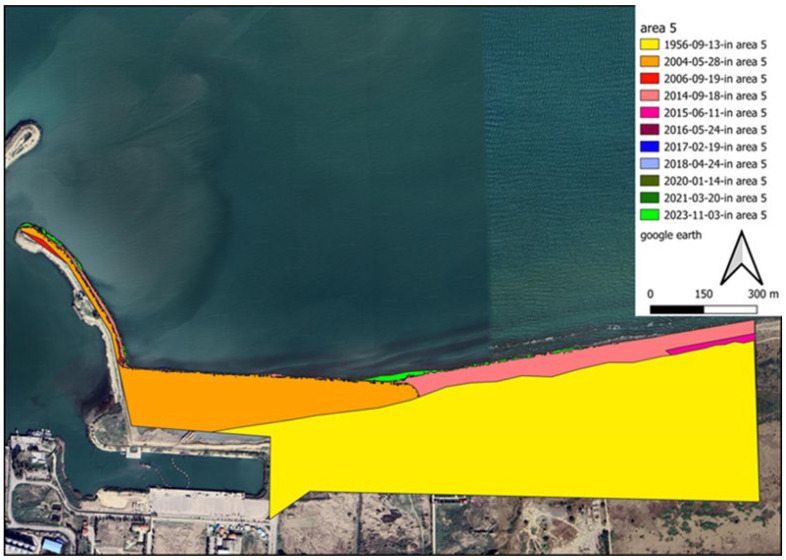
Changes on the right side of the eastern breakwater arm.

Owing to changes in the wave direction, part of the sediment load did not enter the harbour basin. Sediments were partially transported near the structure downstream, as depicted in Figure 17, with the sediment that passed the port entrance settling among the port’s eastern breakwater stones, contributing to the breakwater width.

However, it is crucial to note that when a large volume of longshore sediment load is entrapped, a current without sediment is created downstream of the structure. Erosion is an inherent characteristic of this flow process. Hence, in the first years of operation, the shoreline erosion phenomenon is observable beside the breakwater eastern arm. This phenomenon, however, is rather limited, and it is expected to stabilise after a few years.

### 4.2. Identifying the Changes in Shoreline Position Caused by Sediment Transport

**Figure 18:**
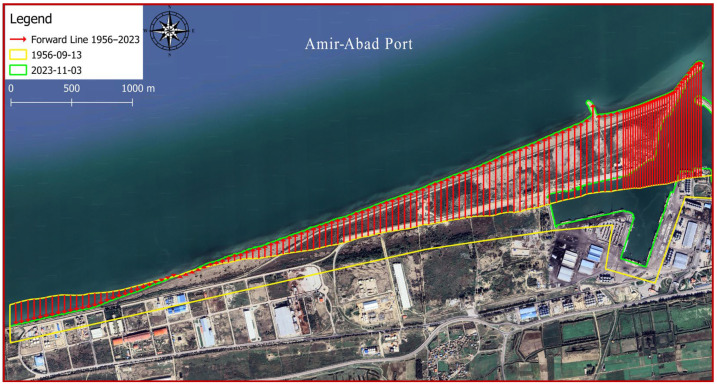
Changes in the location of the shoreline between an intact state and year 2023.

**Figure 19:**
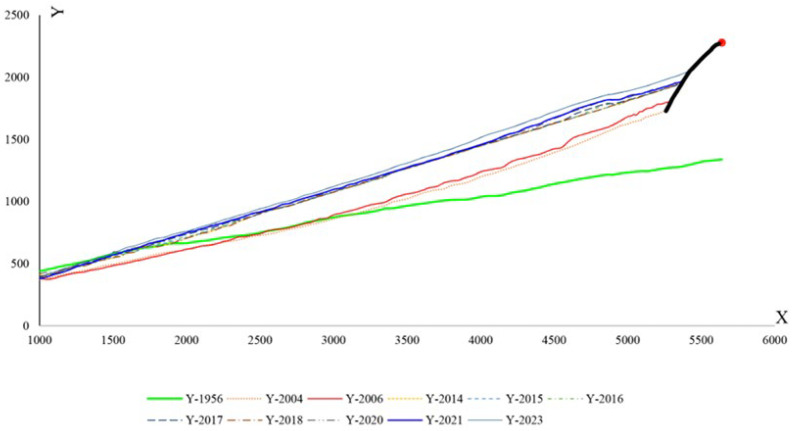
Projections for the coastline’s evolution.

Figure 18 depicts the shoreline positions in 1956 and 2023. Additionally, Figure 19 illustrates the shoreline obtained from satellite images from different years. The reference point is located at (705947.1997, 4080209.9706). For instance, the displayed line in Figure 19 with the colour code Y-1956 indicates the location of the shoreline in the year 1956 in the upstream port (sediment accumulation reservoir). As shown in Figure 18, the trend in the shoreline changes generally agrees with the expected pattern for the direction of the longshore sediment transport (west to east). According to the shoreline changes in subdomains 2 and 3, the shoreline is being displaced towards the sea because of sediment accumulation. Following the real conditions of the shore in the present study does not contradict other methods so that the overall schematic of the configuration of the sedimentation section is similar to the predicted curve in empirical relations such as CERC. As well, the rate of change is noticeable in subdomain 1 after 2014. As a result, port owners should exercise caution when developing depots or infrastructure in this region.

It is also advantageous to quantify the position of the shoreline over time. A measure of the quality of a design can be evaluated by monitoring the entrance of sediment into the port basin and observing the changes in the shoreline position over time. The principal arm of the breakwater appears to be insufficiently long in the target port. From a marine engineering point of view, the main arm of the breakwater needs to exceed the surf zone, which has the highest potential for sediment transport. In addition, the general trend of coast development in region 3 indicates that the rubble-mound element in the sediment accumulation reservoir had no effect on shoreline changes (Figure 19). The addition of this extra element at the breakwater roundhead, which completely exceeds the surf zone, would have been a very effective measure to ensure the port’s continual operation.

**Figure 20:**
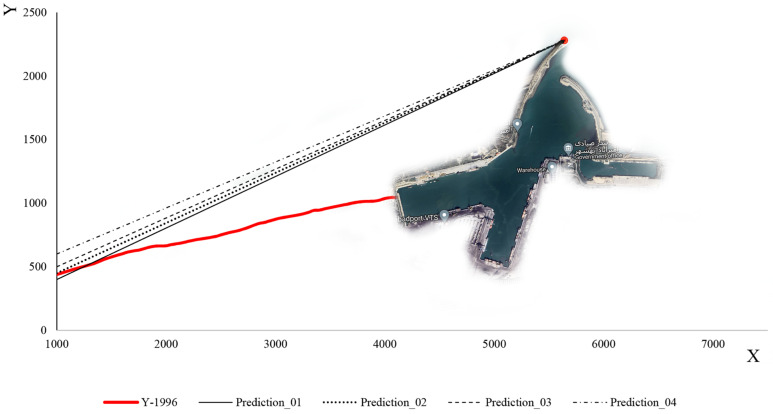
Coastline predictions referring to the breakwater arm.

Different polynomial trend functions are assigned to each of these shorelines based on their displacement. By allocating a linear trend line, R^2^ ≥ 0.98 was calculated. It can be concluded that the shoreline configuration that may reach the breakwaters roundhead follows a linear-type one (Figure 20). The shoreline begins to advance seaward as sediment accumulates behind the main breakwater arm of the harbour. This process continues until the shoreline reaches the breakwater’s roundhead. Consequently, the port’s performance will be adversely affected, and its lifespan is approaching its end. When this disaster occurs, the shoreline meets the breakwater roundhead at one end; however, at the other end, there is no known location upstream of the harbour. For the second end of the shoreline, four different states are being considered in order to solve this problem. Figure 20 illustrates these four states by the labels Prediction_01 to Prediction_04. The ANNs were trained to predict how long after the harbour’s construction each of these states will occur. The results of these predictions are presented in Table 4.

Table 4 shows the coordinates of the breakwaters roundhead point in the local coordinate system in the second column. In the third column, the coordinates of the unknown end of the shoreline at the time of the failure of the port are shown in each scenario. The fourth column illustrates how long it will take for the port to fail due to sediment accumulation from construction. Table 4 indicates that the region behind the principal arm will be completely filled by sediment accumulation in six years if the “Prediction_01” pattern is followed. The sediment reservoir will, however, be completed within about ten years if the “Prediction_04” pattern is followed. Hence, it is noted that if port managers do not take any action, the port’s performance will decrease because of sediment accumulation in six to ten years.

## 5. Conclusions

Located near the borderline between southern and eastern countries in the Caspian Sea, the Amir-Abad port plays an essential commercial role for its users. The simultaneous operation of this port, which is being confronted with longshore sedimentation, and the development of surrounding structures that have altered the hydrodynamic regimen has greatly impacted its performance. Despite the significance of this issue, there have been few investigations conducted in this region. The focus of these studies was on one of the most effective factors. However, in this study, changes in coast area and shoreline position are evaluated based on high-resolution satellite images between 2004 and 2023. An SVM method was used to classify areas in satellite images into two categories, dry and wet. In order to evaluate dynamic changes in the region’s shoreline, categorized images from different years were compared. The following is a summary of the results:

- Satellite images were interpreted for various time periods to determine and report on the areas of coastal growth. In the period 1956 to 2014, the area increased by approximately 61,000 m^2^/y. By developing an industrial region upstream of the port and constructing protective structures to control sediment flow, the amount of sediment transported to the port is reduced. In the region of the sediment accumulation reservoir, the coast’s growth has reached approximately 49,000 m^2^/y.
- The patterns in coastline changes are plotted and reported in terms of time by creating a local Cartesian coordinate system in the port upstream and digitising the shoreline positions during different years. The results of these studies are not limited to reporting past events. An artificial neural network was used to analyse the patterns of the shoreline position changes in the port sediment accumulation region based on these results.
- The general trend of shoreline alterations over the examined period of time indicates that the length of the principal arm of the breakwater is not sufficient for the current environmental conditions. In the area of sediment accumulation, port managers constructed an additional arm, but this structure does not affect coastline formation and it appears that this decision was not appropriate.
- In the local coordinate system, the patterns of each shoreline over time were similar to a high degree of accuracy. A trained artificial neural network has shown that 6 to 10 years of life for the port should be considered when predicting potential shoreline formation. At this point, the coast has reached the principal breakwaters roundhead.
- The use of satellite images and the examination of actual conditions in the study domain leads to results that take into account both natural and human phenomena. Mathematical models cannot incorporate all of these factors, since the driven equations cannot be solved. Consequently, combining satellite images with artificial neural networks in the current study may be an effective method that does not simplify assumptions.

Several potential directions have been identified for future work to enhance and expand upon in the context of the Amir-Abad port, as follows:

1. Integration of Additional Data Sources: incorporating data from other high-resolution satellite imagery and aerial surveys can provide a more comprehensive understanding of coastline dynamics and enhance classification accuracy.
2. Advanced Machine Learning Techniques: exploring and comparing the performance of other advanced machine learning algorithms, such as convolutional neural networks (CNNs) and Random Forests, can further refine the classification of land and water classes.
3. Climate Change Impact Analysis: investigating the influence of climate change factors, such as sea level rise and increased storm frequency, on observed coastline changes can provide valuable insights for coastal management and planning.
4. Long-Term Monitoring and Prediction: developing a predictive model using time series analysis to forecast future coastline changes and assess the potential impacts on the region’s infrastructure and ecosystems.
5. Community Engagement and Policy Development: engaging with local communities and policymakers to share findings and collaboratively develop strategies for sustainable coastal development and conservation.

## Figures and Tables

**Table 1:** Date and source of the high-resolution satellite images, along with their approximate spatial resolution.

Date of Acquisition	Source	Approximate Image Resolution (m)
1956.13.09	USGS Aerial Photo	1.9–2
2004.28.05	Digital Globe	1.2–1.5
2006.19.09	Digital Globe	1.2–1.5
2014.18.09	CNES/Astrium	1.2–1.5
2015.11.06	Digital Globe	1.2–1.5
2016.24.05	Digital Globe	1.2–1.5
2017.23.10	CNES/Astrium	1.2–1.5
2018.24.04	CNES/Astrium	1.2–1.5
2020.14.01	Digital Globe	1.2–1.5
2021.20.03	Digital Globe	1.2–1.5
2023.03.11	CNES/Astrium	1.2–1.5

**Table 2:** Error values for the classification of the images used in the present investigation.

Date of Acquisition	Overall Accuracy	Kappa Coefficient
1956.13.09	92.18	0.916
2004.28.05	96.14	0.939
2006.19.09	96.35	0.959
2014.18.09	96.60	0.951
2015.11.06	96.22	0.942
2016.24.05	96.90	0.959
2017.23.10	96.78	0.954
2018.24.04	96.53	0.948
2020.14.01	96.81	0.955
2021.20.03	96.87	0.956
2023.03.11	96.92	0.959

**Table 3:** Increment/decrement in the shore area in subdomains 2 and 3.

Period	Area Variation (m^2^)
1956–2004	378,346
2004–2006	106,654
2006–2014	614,430
2014–2015	−1953
2015–2016	17,842
2016–2017	26,768
2017–2018	1436
2018–2020	41,926
2020–2021	19,542
2021–2023	134,467

**Table 4:** Predicted time of occurrence for the shoreline position.

I.D	First Point Coordinates	End Point Coordinates	Estimated Lifetime
Prediction_01	(1000, 400)	(5640, 2280)	Δt = 33.33 y → 2030
Prediction_02	(1000, 450)	(5640, 2280)	Δt = 36.31 y → 2033
Prediction_03	(1000, 500)	(5640, 2280)	Δt = 37.04 y → 2034
Prediction_04	(1000, 600)	(5640, 2280)	Δt = 37.45 y → 2034

## Data Availability

All satellite images used in this research are available via Google Earth Pro software and the USGS website (https://earthexplorer.usgs.gov/). Pre-processed images can be requested upon request.
